# Application of the Phage Lysin Ply5218 in the Treatment of *Streptococcus suis* Infection in Piglets

**DOI:** 10.3390/v11080715

**Published:** 2019-08-05

**Authors:** Zhaofei Wang, Jingjiao Ma, Jian Wang, Denghui Yang, Licheng Kong, Qiang Fu, Yuqiang Cheng, Hengan Wang, Yaxian Yan, Jianhe Sun

**Affiliations:** 1 Shanghai Key Laboratory of Veterinary Biotechnology, Key Laboratory of Urban Agriculture (South), Ministry of Agriculture, School of Agriculture and Biology, Shanghai Jiao Tong University, Shanghai 200240, China; 2 Shanghai Animal Disease Control Centre, Shanghai 201103, China

**Keywords:** *Streptococcus suis*, lysin, piglet, infection, treatment

## Abstract

*Streptococcus suis* (*S. suis*) is a gram-positive bacterium and zoonotic pathogen. Currently it poses a serious problem in the swine industry due to the emergence of antibiotic-resistant bacteria. Thus, novel antimicrobials against *S. suis* infections are urgently needed. In the previous study, a cell wall hydrolase or lysin derived from Streptococcus prophage phi5218, termed Ply5218, was identified. This lysin showed strong bacteriolytic activity against *S. suis*. In the current study, the in vitro data showed that after incubation with pig serum, the bacteriolytic efficacy of Ply5218 declined in a time-dependent manner. The in vivo assays indicated that a Ply5218 triple treatment (6, 24, and 48 h post infection) was effective against various serotypes of *S. suis* in a murine infection model. This regimen also alleviated streptococcal-induced clinical symptoms in piglets and significantly reduced the bacterial burden and levels of interleukin 6, a proinflammatory cytokine. This study indicates that Ply5218 shows strong antibacterial activity in pigs and has the potential to be used as a treatment for infectious diseases caused by *S. suis*.

## 1. Introduction

*Streptococcus suis* serotype 2 (SS2) is a major zoonotic pathogen worldwide, being the causative agent of meningitis, arthritis, endocarditis, sepsis, pneumonia, and sudden death in both swine and humans [1,2]. *S. suis* causes persistent infections in pigs, and the pathogen can be found in the nostrils, tonsils, and genital tracts of healthy pigs [1,2,3,4]. Thus far, 33 serotypes have been identified, among which SS2 is the most frequently reported and the most virulent serotype worldwide [1,5]. In swine, SS2 causes severe multiorgan infections which requires complex treatment. Without timely treatment, SS2 infections in humans may be fatal [6]. In the past, the treatment of SS2 infections mainly depended on the use of antibiotics [7]. However, overuse of antibiotics and the emergence of bacterial multidrug resistance in recent years now pose a major challenge to the treatment of clinical SS2 infections worldwide [8,9,10].

Given the resistance to traditional antibiotics, with the lagging of developing new antibiotics, researchers are focusing on the potential of bacteriophages, commonly known as phages, as alternative antimicrobial agents [11,12,13]. Lysins are phage encoded peptidoglycan hydrolases that act at the end of viral infection to lyse bacterial host for virion progeny release [14,15]. Lysins contribute to the powerful bactericidal effect of phages via digestion of the bacterial cell wall peptidoglycan [16]. Compared to conventional antimicrobial chemotherapy, lysin therapies may present several advantages: (a) lysin can specifically lyse target pathogens without disturbing commensal flora; (b) to date, bacterial resistance to lysins has not been reported, as lysins directly target the peptidoglycan network that is crucial for bacterial viability and fitness; and (c) some lysins destabilize bacterial biofilms [16,17]. Thus, the potential of lysins as an antimicrobial agent has been investigated for various biomedical applications, including food science and microbiological diagnostics [17,18,19]. Their use for the treatment of multidrug-resistant bacterial infections in animal models has been intensively studied [18,20,21] and a few clinical trials have demonstrated that phage lysins have enormous potential to be used as therapeutics in humans [22,23].

Previous studies investigated the efficacy of different phage lysins against Streptococcus-induced infections in murine models [24,25,26,27]. However, the bacteriolytic effect of lysin targeting serotype SS2 has not been studied in piglets, which are natural host of SS2. In our previous study, we identified a novel *S. suis* lysin, Ply5218, and this lysin exhibited broad spectrum activity against various serotypes of *S. suis* [27]. In the present study, we investigated the efficacy of Ply5218 in piglets, thereby highlighting its therapeutic potential to control *S. suis* infection in these animals.

## 2. Materials and Methods

### 2.1. Bacterial Strains and Growth Conditions

The SS2 strain-HA9801 was kindly provided by Professor Chengping Lu, Nanjing Agricultural University, China. The *Streptococcus suis* serotype 7 (SS7) strains-SS-7 and serotype 9 (SS9) strains-SS-9 were stored in our laboratory which have been isolated from diseased pigs between 1998 and 2005 in China [28]. All of the *S. suis* strains were cultured aerobically in brain heart infusion (BHI) broth in a shaker (180 rpm) at 37 °C. The bacterial burden, given as the number of CFUs of *S. suis*, was measured in BHI agar supplemented with 5% (vol/vol) fresh sheep blood under static conditions at 37 °C. The *Escherichia coli* (*E. coli*) strains used for gene cloning and production of recombinant proteins were grown in Luria-Bertani (LB) broth with shaking at 37 °C.

### 2.2. Expression and Purification of Ply5218

The Ply5218 lysin was characterized previously in our laboratory and shown to lyse *S. suis* efficiently. The expression and purification of Ply5218 were carried out as described previously, with some modifications [20,27]. Briefly, the Ply5218 gene of prophage phi5218 (accession number: KC348600.1) was amplified by PCR using primers Ply5218-*Bam*HI (5′- CCGGGATCCATGAGTAATCTAGGACTTAAAATG-3′) and Ply5218-*Xho*I (5′-GGACTCGAGCTATTTTACATTGCCCCAC-3′) and cloned into the expression vector pET-28a (containing His-tag) to obtain pET28a-Ply5218. The recombinant expression vector pET28a-Ply5218 was then transformed into *E. coli* BL21 (DE3) competent cells. The positive clones were identified by specific PCR. The bacterial cells expressing Ply5218 were then cultured in the LB broth at 37 °C until the optical density at 600 nm (OD600) was 0.6. After cooling, 1 mM isopropyl-β-D-thiogalactoside was added to induce the production of Ply5218 and then put into a shaker at 27 °C for 5 h. The induced cells were washed, resuspended, and homogenized using a sonicator. After centrifugation, the protein was purified by affinity chromatography with Ni-NTA columns (GE Healthcare BioSciences, Pittsburgh, PA, USA). To obtain a recombinant protein for use in the animal experiments, endotoxin was removed using phosphate-buffered saline (PBS) containing 0.1% Triton X-114 (Sigma-Aldrich, St. Louis, MO, USA) to elute protein from Ni-NTA columns [29].

### 2.3. Determination of the Minimum Enzyme Concentration (MEC) of Ply5218 In Vitro

To determine the minimum concentration for the activity of Ply5218 against *S. suis*, its MEC was measured using a 96-well plate assay, as described by Nelson et al., with minor modifications [30]. Briefly, the *S. suis* strains SS2, SS7, and SS9 were freshly cultured in a shaker at 37 °C and adjusted to an OD600 of 0.6 (3 × 10^8^ CFU/mL) with PBS as the target bacteria, respectively. The MEC of Ply5218 was monitored by adding 100 µL of the bacterial suspension to 100 µL of Ply5218 at different final concentrations (1.25, 2.5, 5, 10, 20, and 40 µg/mL) on a 96-well microtiter plate. The OD600 values were monitored with incubation time of 15, 30, 45, and 60 min.

### 2.4. Impact of Piglet Serum on Ply5218 Activity

To determine the lytic activity of Ply5218 in pig blood serum, a 96-well plate assay in vitro was performed. In the assay, 10 µL of the Ply5218 at 100 µg/mL were mixed with 90 µL of serum collected from healthy piglets, followed by incubation at 37 °C for 0.5, 1, 2, 6, 12, 18, 24, 36, and 48 h. As a positive control, the same amount of the Ply5218 was mixed with 90 µL of PBS, followed by incubation at 37 °C for 48 h. Subsequently, the mixture was incubated with 100 µL of exponentially growing cells of *S. suis* adjusted to an OD600 of 2.0 (1 × 10^9^ CFU/mL) with PBS in a 96-well plate at 37 °C. The OD600 values were monitored with an incubation time of 30 min. The turbidity was calculated by the OD600 after 30 min/original OD600 of the mixture, indicating the bacterial concentration after incubation.

### 2.5. Murine Model of S. suis Infection Treated with Ply5218

Female BALB/c 6-week-old mice were purchased from the Experimental Animal Center, Shanghai Jiao Tong University. The *S. suis* strains SS2, SS7, and SS9 were cultured in BHI broth to exponential phase (about 1 × 10^9^ CFU/mL) in a shaker at 37 °C, harvested by centrifugation at 5000× *g* for 5 min, washed 3 times in PBS, and resuspended in PBS, respectively. The mice were randomly divided into different groups, with sixteen mice in each group (ten mice were for survival rate and six mice were tested for bacterial burden). A lethal dose (5 × 10^8^ CFU/mouse) of SS2, SS7, or SS9 was injected unilaterally into the abdominal cavities of the mice, and Ply5218 (150 µg) was injected once into the other side either at 0, 1, or 2 h (150 µg) post infection, respectively. In a separate group (triple treatment group) the mice were treated with Ply5218 at 6, 24, and 48 h post infection (50 µg each time). The infected mice treated with PBS were served as an untreated control group. Mice were observed for 7 d post infection. The survival rates were calculated with untreated, 0 hours post infection (hpi), triple, and uninfected groups. The CFU burden in blood (facial vein) and spleen were determined at 2 and 4 d post infection of all groups. Each spleen was homogenized in 1 mL of PBS, and the homogenized spleen or blood was 10-time serially diluted in sterile PBS for quantitative culturing on a BHI agar plates. All cultures were incubated (37 °C) for 24 h, and the resulting colonies were enumerated as CFU/g (for spleen) or CFU/mL (for blood).

### 2.6. Piglet Model of S. suis Infection Treated with Ply5218

Fourteen 10-week-old piglets with no history of *S. suis* infection were purchased from Hangtou pig breeding (nursery) farm (Shanghai, China). After 1 week adaptation, the piglets were randomly divided into three groups, with four piglets in each group, and one mock control group (*n* = 2). Both challenge and treatment were applied intramuscularly. The piglets in the challenge groups were inoculated with the HA9801 strain (recovered from piglet) suspended in 10 mL of PBS (5 × 10^9^ CFU per piglet) by an intramuscular injection on the left-hand side of the neck, and Ply5218 or PBS were inoculated by an intramuscular injection on the right-hand side of the neck. After inoculation, the following doses and regime of the lysin or PBS were administered to the piglets:

Group 1 (*n* = 4): Purified lysin (450 µg/kg) was administered with three doses of 150 µg/kg at 6, 24, and 48 h post bacterial challenge (Triple treatment group).

Group 2 (*n* = 4): PBS (lysin equivalent volume) was given at 6, 24 and 48 h post bacterial challenge (Untreated group).

Group 3 (*n* = 4): Purified lysin (450 µg/kg) was administered at 6, 24, and 48 h with three doses of 150 µg/kg to uninfected piglets (Uninfected group).

Group 4 (*n* = 2): PBS was administered at each side of piglet’s neck (bacteria and lysin equivalent volumes, respectively) as a control (Uninfected and untreated group).

During the study, the body temperatures (rectal) of the animals and clinical symptoms were recorded daily until 7 d post infection. Clinical symptoms including impaired mental states and claudication were scored. Scores of 0 to 3 were used, where 0 = normal; 1 = mild depression and signs of lack of coordination visible only after manipulation of the piglet; 2 = moderate depression and obvious signs of a lack of coordination; and 3 = severe depression and signs of asynergia or lethargy. Locomotive disorder symptoms were scored as follows: 0 = normal, 1 = the animal avoided movement on the leg, and 2 = the animal was reluctant to stand. Recovery was considered as a rectal temperature below 39.5 °C, as well as both mental state and locomotive disorder scores ≤ 1.

The bacterial burdens in blood collected from the anterior vena cava of the piglets were examined. Serum levels of proinflammatory cytokines were also determined. To test the CFU counts, blood was 10-times serially diluted in sterile PBS for quantitative culturing on BHI agar plates. All cultures were incubated (37 °C) for 24 h, and the colonies were enumerated as CFU/mL. Commercial enzyme-linked immunosorbent assay (ELISA) kits (Abcam, Cambridge, UK) were used to evaluate the serum levels of the proinflammatory cytokine interleukin 6 (IL-6) post infection and after Ply5218 treatment. The cytokine levels were measured in accordance with the manufacturer’s instructions.

### 2.7. Ethical Statement

All the animal experiments were approved at 03/01/2017 by the Institutional Animal Care and Use Committee of Shanghai Jiao Tong University (Approval no. 20170103) and were performed in accordance with the Animal Care and Use Guidelines made by the Ministry of Science and Technology of China.

### 2.8. Statistical Analysis

For all experiments, data points were plotted using GraphPad Prism (v. 7.01) software (GraphPad Software, Inc., San Diego, CA, USA). The data are presented as mean values ± standard errors of the means (SEM). For the analysis of the data obtained in the experiments, the nonparametric Mann–Whitney *U* test was used. A *P* value of < 0.05 was considered significant.

## 3. Results

### 3.1. Expression, Purification and In Vitro MEC of Ply5218

**Figure 1:**
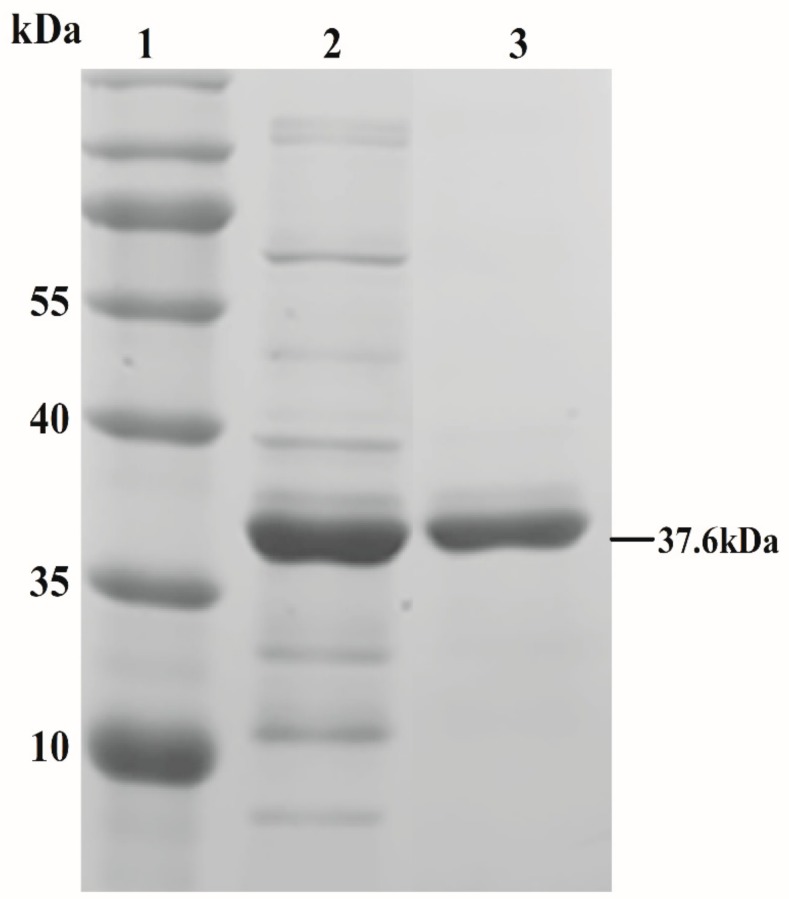
Analysis of purified lysin from *Streptococcus* prophage phi5218 (Ply5218) by sodium dodecyl sulfate polyacrylamide gel electrophoresis (SDS-PAGE) assay and Coomassie brilliant blue staining. Lane 1, ladder; lane 2, crude protein extracted from IPTG-induced *E. coli* BL21 (DE3) cells producing Ply5218; lane 3, purified Ply5218 protein (37.6 kDa, 31.1 kDa of Ply5218 plus 6.5 kDa of tag in pET 28a).

**Figure 2:**
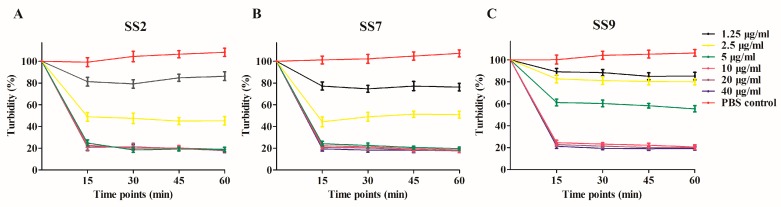
The turbidity reduction of *Streptococcus suis* (**A)** *S. suis* serotype 2 (SS2), (**B**) *S. suis* serotype 7 (SS7), and (**C**) *S. Suis* serotype 9 (SS9) after treatment with the indicated concentrations of Ply5218. The data shown are means ± SEMs for at least three independent experiments.

Ply5218 was successfully produced in *E. coli* BL-21 (DE3). The purified Ply5218 protein migrated as 37.6 kDa polypeptide in sodium dodecyl sulfate polyacrylamide gel electrophoresis (SDS-PAGE) (Figure 1). The purified protein concentration was 3.43 mg/mL. A turbidity reduction assay was performed to examine the MEC of the Ply5218 protein against *S. suis*. The results revealed a significant decrease in activity within 15 min, and there was no observable change within the next 60 min. The MEC of Ply5218 against SS2, SS7, and SS9 was 5 µg/mL, 5 µg/mL, and 10 µg/mL, respectively (Figure 2). The MEC of Ply5218 was higher than that found in a previous study [27]. This might be due to the lysin being purified before the test by Triton X-114 and then eluted from Ni-NTA columns.

### 3.2. Impact of Piglet Blood Serum on Lytic Activity of Ply5218

**Figure 3:**
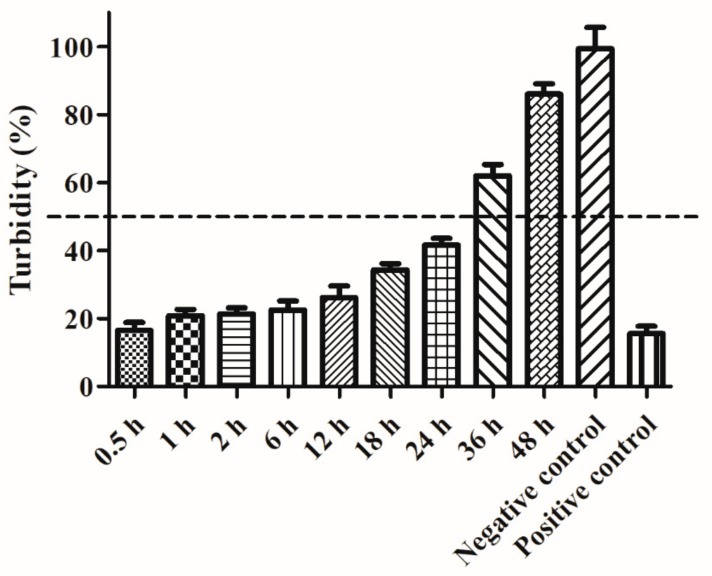
Turbidity (the OD600 after 30 min/original OD600) reduction of *S. suis* cell suspensions (strain HA9801) after preincubation of Ply5218 in piglet serum. Serum was collected from healthy piglets and incubated with Ply5218 at 37 °C. Turbidity reduction of HA9801 treated with the Ply5218/serum mixture was measured after 30 min incubation. The data shown are means ± SEMs from at least three independent experiments.

To study the effect of pig serum on Ply5218 activity, serum was collected from healthy piglets and incubated with Ply5218 for different periods at 37 °C. As shown in Figure 3, the endolysin activity slowly decreased during its incubation in serum. Decline of the enzyme lytic capacity was particularly evident after 24 h incubation. The bacterial turbidity (the OD600 after 30 min/original OD600) was less than 50% within the first 24 h of incubation but more than 50% after 24 h of incubation. These data indicated that the bacteriolytic effect of Ply5218 in pig serum appeared to decline after 24 h.

### 3.3. Application of Ply5218 in the Treatment of S. suis Infection in Mice

**Figure 4:**
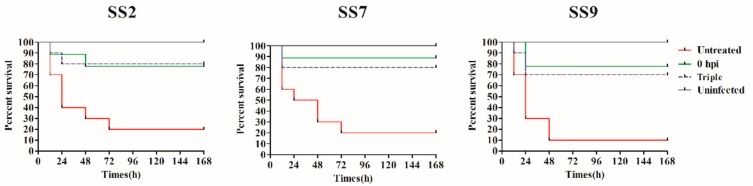
Survival curves of mice infected with *S. suis* and treated with Ply5218. A lethal dose (5 × 10^8^ CFU/mouse) of SS2, SS7 or SS9 was injected into the abdominal cavities of the mice, and Ply5218/PBS on the other side. In one group, Ply5218 was administrated immediately (0 h) post infection. In the other treatment group, mice were treated with Ply5218 at 6, 24, and 48 h post infection (triple treatment group). The infected mice treated with phosphate-buffered saline (PBS) served as an untreated group. The uninfected mice treated with Ply5218 served as an uninfected group. hpi: hours post infection.

**Figure 5:**
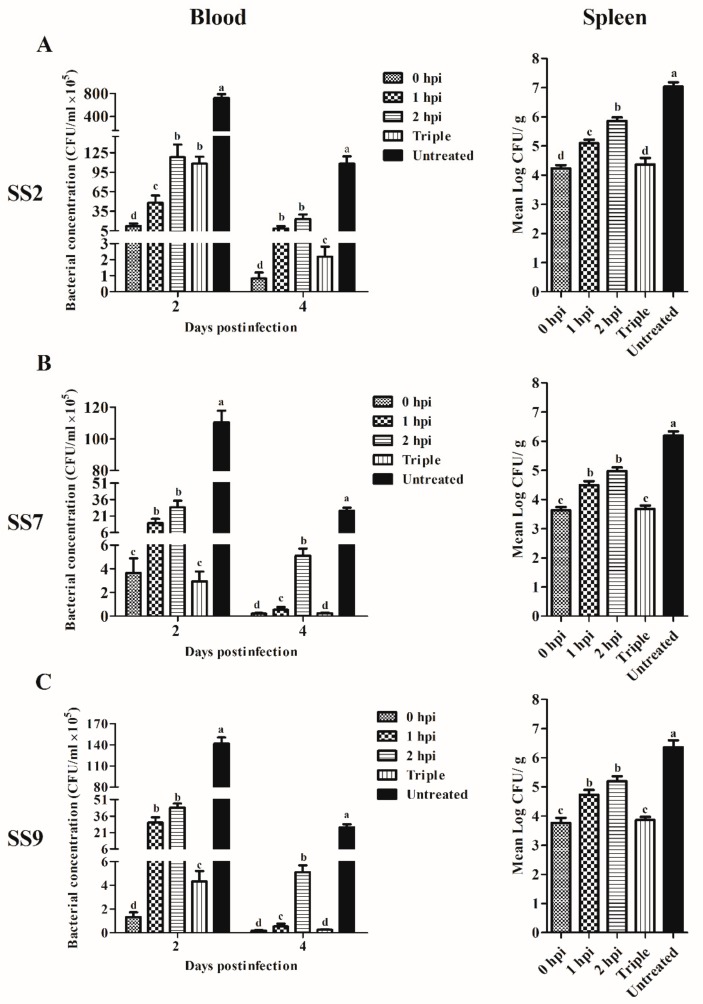
The bacterial burden in murine blood and spleen (2 and 4 d post infection) following bacterial challenge with SS2 (**A**), SS7 (**B**), and SS9 (**C**). Ply5218 was administered at 0, 1, or 2 h post infection. In the triple treatment group, mice were treated with Ply5218 at 6, 24, and 48 h post infection. PBS was administered to infected mice in the untreated group. The results are shown as means ± SEM from triplicate experiments. For each sampling time, means with different letters on a given day are statistically different (*p* < 0.05).

Prior to study the therapeutic efficacy of Ply5218 in piglets, we studied the effect of Ply5218 in mice to optimize the therapeutic dosage. The results showed significantly higher survival rates for the mice treated at 0 h (80% for mice infected with SS2, 90% for mice infected with SS7, and 80% for mice infected with SS9) and the mice receiving the triple treatment (80% for mice infected with SS2, 80% for mice infected with SS7 and 70% for mice infected with SS9) than that of the control groups (20% for SS2-infected mice, 20% for SS7-infected mice, and 10% for SS9-infected mice) after 7 days post infection (Figure 4). To examine the bacterial load in the organs and blood of mice after Ply5218 therapy, the mice were sacrificed at 2 and 4 d post infection. The results showed that the bacterial burden in blood of all the Ply5218-treated mice was significantly lower than that in the Ply5218-untreated control mice at 2 and 4 d post infection (Figure 5). At four days post infection, regardless of whether the mice were infected with SS2, SS7, or SS9, the bacterial burden in both the triple treatment group and the treatment at 0 h group was significantly lower than that of the blood and spleen of the groups treated at 1 and 2 h post infection (Figure 5). These findings indicated that triple or immediate treatment with Ply5218 effectively antagonized murine bacteremia caused by different serotypes of *S. suis*.

### 3.4. Application of Ply5218 in the Treatment of S. suis Infection in Piglets

**Figure 6:**
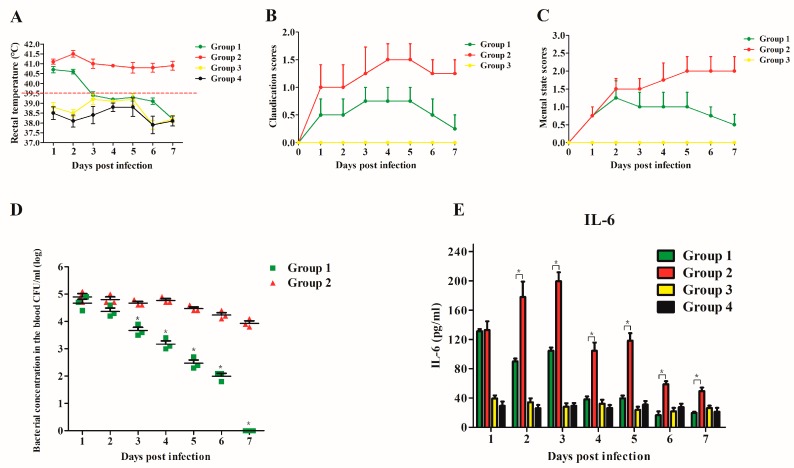
Rectal temperature (**A**), clinical scores for claudication (**B**), mental status (**C**), bacterial load (**D**), and proinflammatory cytokine (**E**), levels in blood or serum of piglets in different treatment groups after challenge with *S. suis* strain HA9801. Group 1, triple treatment after bacterial inoculation (triple treatment group); group 2, bacterial challenge and PBS treatment (untreated group); group 3, Ply5218 administration with no bacterial challenge (uninfected group); and group 4, uninfected and untreated piglets. Ten milliliters of blood were collected from each piglet at different time points after the treatment and assayed for bacterial burden or IL-6 (sera) using commercial ELISA kits. In D and E, the asterisks indicate that both the CFU counts and the serum level of IL-6 in triple treatment group (group 1) were significantly lower (*p* < 0.05) than that in group 2 (untreated), respectively. Signs of infection and mental status were monitored for 7 d post infection. Recovery was considered as a rectal temperature below 39.5 °C, as well as both mental state and claudication symptom scores ≤1. Samples collected from each piglet were tested in triplicates. The data shown are means ± SEMs.

No clinical signs of *S. suis* infection were observed in piglets during the adaptation period prior to challenge. All the piglets in the untreated group (group 2) showed obvious clinical symptoms of the infection caused by *S.suis*, such as fever (higher than 40 °C through 7 days), an obvious lack of coordination, and severe claudication (Figure 6A–C). In contrast, in the triple treatment group (group 1), the piglets showed fewer clinical signs, such as fever until the third day of the treatment. In addition, the health status of the piglets in group 1 had returned to almost normal at 7 d post infection.

The bacterial burden in blood samples of the triple treatment group (group 1) was significantly reduced as compared with that in the untreated (group 2). Furthermore, the CFU counts decreased drastically during the treatment in group 1 (Figure 6D). The bacterium was no longer detectable 7 d post infection in this group. In contrast, CFU counts in the untreated (group 2) remained detectable until the end of the study (Figure 6D). The level of the proinflammatory cytokine IL-6 in group 2 increased during the first 3 d post infection and then started to decrease after day 4 (Figure 6E). Meanwhile, the level of IL-6 in the triple treatment group decreased throughout the study. Compared to group 2, the level of IL-6 in group 1 was significantly lower at most post infection days. The level of IL-6 in the lysin only group (group 3) were similar to those in the control group (group 4, uninfected and untreated).

## 4. Discussion

SS2 is the most virulent serotype of *S. suis* [1]. Antibiotics have been the standard treatment for SS2 infection for many years. The large-scale use of antibiotics in humans and animals has placed selective pressure on pathogenic microorganisms, including Streptococcus species, and led to drug-resistant *S. suis* [8,9]. Thus, interest has grown in the potential of phage lysins as novel antimicrobials [31,32,33]. Only a few lysins (Ly7917, Ply5218, and LySMP) that specifically targeted SS2 have been reported [27,28,34]. Among those, Ply5218 showed the highest lytic activity and efficacy against SS2 in vitro [27]. Since no study has focused on the application of this lysin against SS2 infections in pigs, the present study explored the potential of Ply5218 as an antimicrobial in piglets.

Studies have showed that lysins were effectively against mice infections caused by *S. suis* [20,24]. In this study, murine bacteremia model was used to assess the bactericidal effect of Ply5218. The bacterial burden in murine blood in the group that received the Ply5218 treatment immediately after the bacterial challenge (0 h) was significantly lower than that in the other infected groups (Figure 5). This finding is in accordance with previous studies employing lysins in murine models [35]. These studies found that the lysins activity to eradicate bacteria was the highest when administered shortly after a bacterial challenge. In this context, another study suggested that the superior therapeutic effect of lysins administered directly after a bacterial challenge may be due to their effect on local infections and the prevention of diffusion of bacteria [20,36]. Interestingly, in the present study, mice mortality and bacterial burden were comparable in the triple (50 µg each time) and immediate (150 µg of single dose) treatment groups, being significantly lower when compared with the other groups. This indicates that triple treatment provided efficient protection against streptococcal infections. In this study, the piglets in the triple treatment group received three doses (150 µg/kg each time) of Ply5218 on consecutive days. As expected, the condition of the piglets in this group was better than that in the untreated group, with improved health status, a reduced bacterial load, and lower levels of the proinflammatory cytokine IL-6 (Figure 6).

Previous studies have demonstrated that lysins provide prophylaxis against bacterial infections when administered prior to infection [37,38]. To provide such protection, lysins must remain active in the target animal for a number of days. As shown by the in vitro study, Ply5218 showed bacteriolytic activity after incubation with serum for 24 h (Figure 3). Therefore, Ply5218 appears to remain active in piglets for at least 24 h. However, in humans most proteins smaller than 60-65 kDa tend to be rapidly eliminated by the renal system [39]. Thus, the pretreatment of lysin may not provide solid prophylaxis in vivo.

Side effects are occasionally associated with bactericidal agents. These include the release of large amounts of pathogen-associated molecular patterns, which are recognized by toll-like receptors and result in the induction of proinflammatory cytokines in mammals [40]. In the present study, there was no differences in the expression of proinflammatory cytokine (IL-6) in the uninfected piglets treated with Ply5218 compared to those in the control group throughout the study (Figure 6E). Furthermore, the Ply5218 treatment was not associated with clinical signs of fever, lethargy, or claudication. Therefore, Ply5218 appears to have had no obvious side effects. The researchers believe that, irrespective of the administration route, lysins make the host produce antibodies against lysins, which may affect the bacteriolytic activity of lysins in vivo [16]. However, Zhang et al. suggested that antibodies against lysins may not affect the bacteriolytic efficiency of lysins in vitro or in vivo, as these antibodies may not always bind to the enzymatic activity region of lysins [41]. If lysins are administered within one week of the detection of infection, the antibodies that are produced may be insufficient to influence the lytic activity of lysins. The results of our study support this idea, with Ply5218 maintaining a bacteriolytic effect against *S. suis* in vivo in the triple treatment group (Figure 6D).

The findings of the present study indicate that Ply5218 exhibits lytic activity against various serotypes of *S. suis*. Furthermore, Ply5218 provided protection against SS2, SS7, and SS9 challenge in both mice and piglets. Some caveats need to be considered in terms of the therapeutic efficacy of Ply5218, for example, it must be administered shortly after infection. Nevertheless, Ply5218 shows great potential as an alternative to antibiotics.
